# Hypophysial angiogenesis decodes annual time and underlies physiological adaptation to seasonal changes in the environment

**DOI:** 10.1002/jez.2639

**Published:** 2022-07-18

**Authors:** Domingo J. Tortonese

**Affiliations:** ^1^ Laboratories for Integrative Neuroscience and Endocrinology, Faculty of Health Sciences University of Bristol Bristol UK

**Keywords:** angiogenesis, melatonin, pars tuberalis, photoperiod, pituitary gland, prolactin, VEGE‐A

## Abstract

Adaptation to annual changes in the environment is controlled by hypophysial hormones. In temperate zones, photoperiod is the primary external cue that regulates annual biological cycles and is translated by the pattern of melatonin secretion acting primarily in the hypophysial pars tuberalis. Angiogenic mechanisms within this tissue contribute to decode the melatonin signal through alternative splicing of the vascular endothelial growth factor A (VEGF‐A) gene in both the pars tuberalis and the capillary loops of the infundibulum. The resulting melatonin‐evoked differential productions of VEGF‐A isoforms will induce seasonal remodeling of the vascular connection between the hypothalamus and hypophysis, and act as paracrine messengers in the pars distalis to generate the required seasonal endocrine response. Specifically, the long melatonin signal in winter upregulates antiangiogenic VEGF‐A isoforms, which will reduce the number of vascular loops and the density of VEGF receptors in endocrine and folliculo‐stellate (FS) cells, inhibit prolactin secretion, and stimulate FSH. In contrast, the short melatonin signal in summer upregulates proangiogenic VEGF‐A isoforms that will increase the number of vascular loops and the density of VEGF receptors in endocrine and FS cells, stimulate prolactin secretion, and suppress FSH. A similar system has been identified in long day seasonal breeders, revealing that this is a conserved mechanism of adaptation across species. Thus, an angiogenesis‐based, intrahypophysial system for annual time measurement controls local microvascular plasticity and conveys the photoperiodic signal readout from the melatonin sensitive pars tuberalis to the endocrine cells of the pars distalis to regulate seasonal adaptation to the environment.

## Research Highlights

Angiogenic mechanisms in the hypophysial pars tuberalis decode photoperiod/melatonin signals through differential production of vascular endothelial growth factor‐A isoforms to induce local microvascular remodeling and regulate seasonal endocrine output from the pars distalis.

## Graphical abstract

Intrahypophyseal angiogenic mechanisms translate the photoperiod/melatonin signal to decode annual time for adaptation to seasonal changes in the environment. In the short days of winter (ovine breeding season –BS), the long duration of nocturnal melatonin output stimulates the production of antiangiogenic vascular endothelial growth factor (VEGF‐A) isoforms (VEGF_164/165_b) leading to a reduction in local angiogenesis, suppression of prolactin and stimulation of gonadotrophin secretion; in contrast, in the long days of summer (ovine nonbreeding season –NBS), the short duration of nocturnal melatonin output stimulates the production of proangiogenic VEGF‐A isoforms (VEGF_164/165_) leading to an increase in local angiogenesis, stimulation of prolactin and suppression of gonadotrophin secretion. Adapted from Castle‐Miller et al. (2017).

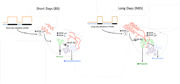

## 1 INTRODUCTION

Successful adaptation of vertebrates to annual changes in the environment requires seasonal changes in the tightly regulated secretion of hypophysial hormones that control an array of body functions such as reproduction, metabolism, body growth, lactation, and the response to stress. The hypophysis (from Greek *hupophusis* ‐undergrowth), or pituitary gland (from Latin *pituitarius* ‐secreting phlegm) lies on the *sella turcica* of the sphenoid bone and comprises two parts of different embryological origin, a glandular part known as the adenohypophysis, of which its largest component is commonly referred to as the anterior pituitary, and a neural part known as the neurohypophysis, of which its major component is also referred to as the posterior pituitary, although, under this classification, the latter also includes the adjacent region of the adenohypophysis—the pars intermedia. From a functional perspective, a key difference between these two parts is that the adenohypophysis secretes hormones synthesized locally, whereas the neurohypophysis releases hormones produced in the hypothalamus. The adenohypophysis comprises three distinctive regions: the pars tuberalis, the pars distalis and the pars intermedia; whereas the neurohypophysis contains fibers that originate in hypothalamic nuclei and includes the neural stalk (composed of the median eminence and the infundibular stem) and the pars nervosa (Green & Harris, 1947). The pars tuberalis surrounds the neural stalk and thus is strategically located between the brain and the rest of the pituitary. The endocrine cells of this region do not strictly match all the cell types found in the pars distalis and this heterogeneity varies across species (Gross, 1984). The five endocrine cell types of the pars distalis (corticotrophs, lactotrophs, somatotrophs, thyrotrophs, and gonadotrophs) produce the six hormones (i.e., adrenocorticotrophin ‐ACTH‐, prolactin, growth hormone ‐GH‐, thyrotrophin ‐TSH‐ and the gonadotrophins luteinising hormone ‐LH‐, and follicle stimulating hormone ‐FSH) that regulate most, but not all, the temporal physiological changes needed to adjust to the predictable annual changes in the environment. The pars intermedia, located between the pars distalis and the pars nervosa, varies in size or degree of development across species and secrets the proopiomelanocortin‐derived α‐melanocyte‐stimulating hormone that regulates, among other features, pigmentation of the integument, whereas the pars nervosa releases oxytocin and vasopressin produced in the supraoptic and paraventricular hypothalamic nuclei to control a range of functions such as parturition, social bonding, water balance and absorption.

The presence of another cell type in the adenohypophysis of agranular, nonendocrine characteristics, provides another putative mechanism of regulation within the gland for adaptation to the environment distinctive from those of the endocrine cells. Folliculo‐stellate (FS) cells, first identified by Rinehart and Farquhar, 1953, and then described as having a star shape and long extensions (Salazar, 1963) are glial‐type, follicle forming cells of the dendritic cell meshwork present in the three regions of the adenohypophysis (Acosta & Mohamed, 2009; Acosta et al., 2010; Henderson et al., 2008), that were shown to form an anastomosing network of channels (Vila‐Porcile, 1972) for communication within the gland through microcirculation via interstitial cavities (Allaerts et al., 1990). Histochemically, these cells can be readily identified by the presence of S100 protein or glial fibrillary acidic protein depending on species (Allaerts & Vankelecom, 2005). FS cells communicate with each other and with endocrine cells in two ways. Through gap junctions FS cells form a long‐range, three‐dimensional system of communication within the pituitary that enables coordinated responses (Fauquier et al., 2001; Morand et al., 1996). Through secretory products, the FS cells control the function of endocrine cells in a paracrine manner (Baes et al., 1987; Denef, 2008; Hentges et al., 2000). Indeed, FS cells have been shown to produce, among other compounds, basic fibroblast growth factor (Ferrara et al., 1987), interleukin‐6 (Vankelecom et al., 1989), nitric oxide (Vankelecom et al., 1997), annexin 1 (John et al., 2004), endocannabinoids (Yasuo et al., 2010) and follistatin (Gospodarowicz et al., 1989), which are important intrapituitary regulators of a variety of key functions such as the response to stress, the differential control of gonadotrophin secretion, and seasonal reproduction (Denef, 2008; Tortonese, 2016; Winters et al., 2007). Critically, FS cells are an important source of vascular endothelial growth factor (VEGF) (Ferrara & Henzel, 1989; Gloddek et al., 1999), and thus play a significant role in the angiogenesis and vascular permeability of the gland. This review focuses on the impact of hypophysial angiogenic mechanisms in the adaptive response of mammals to seasonal changes in the environment and thus on their involvement in annual time measurement.

## 2 HYPOPHYSIAL VASCULAR SUPPLY AND ANGIOGENESIS

The vascular supply to the hypophysis is primarily derived from the anterior (nonprimate species) or superior (primates) hypophysial arteries, which branch from the internal carotid arteries and give rise to an intricate portal system (Popa & Fielding, 1930). Contrary to what it was accepted at the time and using the living toad as the experimental animal model, Bernardo Houssay demonstrated in 1935 that the direction of the bloodflow was from the hypothalamus towards the pituitary (Houssay et al., 1935). It took 14 years for this important discovery, which led Houssay to receiving the Nobel Prize in Physiology and Medicine for the work that followed, to be corroborated in rodents (Green & Harris, 1949). The primary vascular plexus that originates from the hypophysial arteries generates capillary loops with afferent branches that cross the pars tuberalis and penetrate the median eminence/infundibular stalk, and efferent branches that return to the pars tuberalis to then give rise to the long portal vessels that will carry the blood to the pars distalis. The vascular supply to the hypophysis is completed by the posterior (nonprimate species) or inferior (primates) hypophysial arteries, which supply the pars nervosa and give rise to the artery of the lower infundibular stem; this latter vessel generates another vascular plexus that will also provide (via short portal vessels) to the pars intermedia and adjacent region of the pars distalis. As the artery of the lower infundibular stem runs rostrally to reach the primary plexus of the hypophysial stalk, the two plexuses are in communication via this vessel, but, irrespectively of this anatomical feature, the blood supply to the pars distalis in all species is exclusively imparted by veins (Daniel & Pritchard, 1957; Green & Harris, 1947). It is becoming increasingly apparent that the vascular loops of the hypophysial stalk play an even greater role in the seasonal adaptation to a changing environment than conventionally accepted. These vascular loops were first described by Wislocki and King (1936) for the monkey, and then corroborated in a wide range of species such as the cat, dog, rat, guineapig, and rabbit (Green & Harris, 1947; Wislocki, 1937), with the most detailed account of this vascular arrangement being reported by Daniel and Prichard (1957) for the sheep. Although it is well known that the efferent branches of the loops carry hypothalamic stimulatory and inhibitory products from the median eminence to the pars tuberalis and then to the pars distalis to control the secretory activity of endocrine cells, it is important to note that the afferent branches of the loops can first deliver the secretory products of the pars tuberalis to the median eminence; because the afferent branches of the loops make a “u” turn in the median eminence and then continue as efferent branches back to the pars tuberalis (Daniel and Prichard, 1957), the output of the pars tuberalis will return to its point of origin and then will be delivered to the pars distalis via the long portal vessels. This implies that the vascular loops of the hypophysial stalk have the important function of delivering the secretory signals of the pars tuberalis to both the median eminence and the pars distalis.

**Figure 1:**
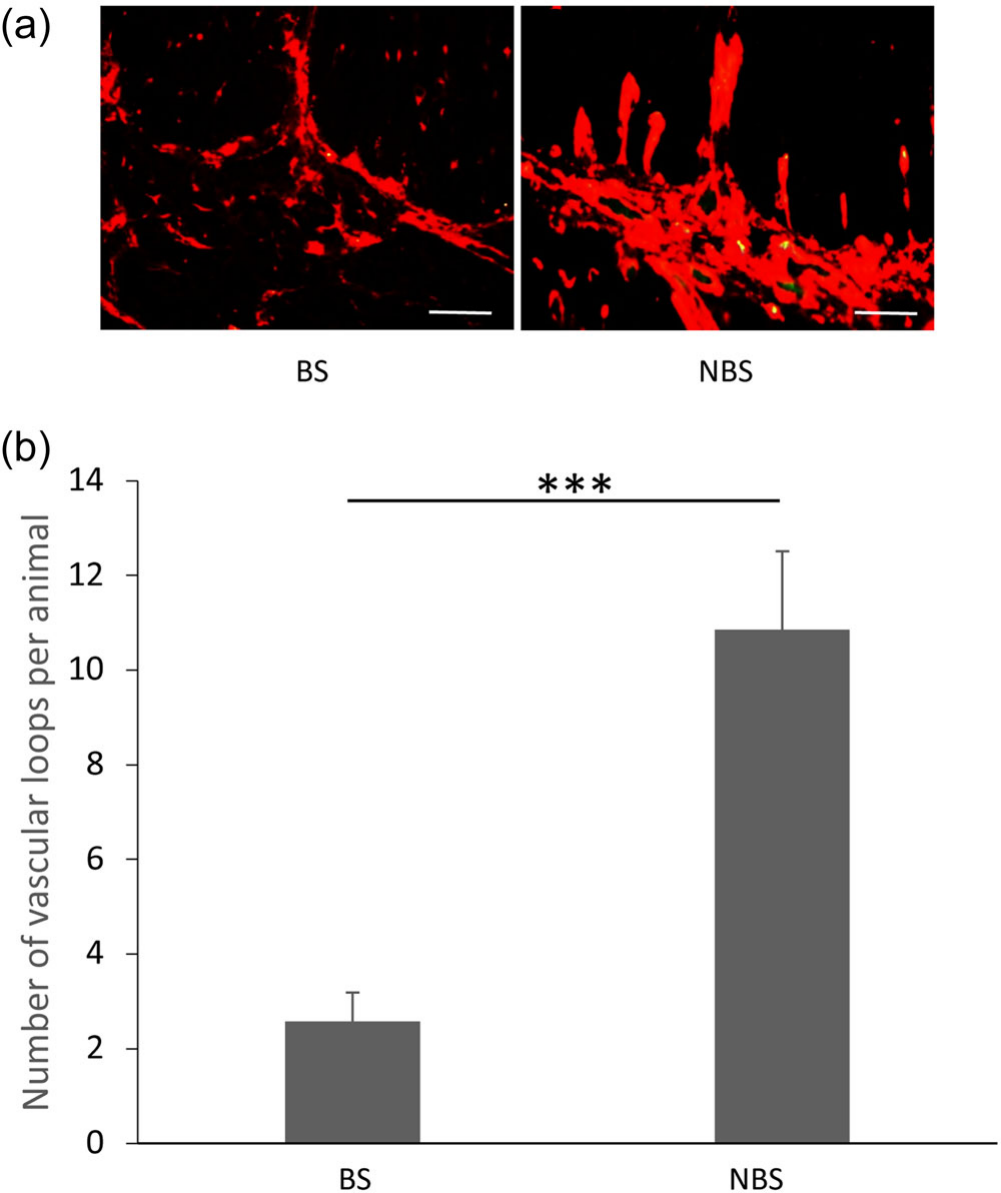
Seasonal microvascular remodeling of the ovine hypophysial stalk (a). Endothelial staining of the vascular loops connecting the pars tuberalis with the infundibulum in sheep during the breeding season (BS ‐winter) and non‐BS (NBS ‐summer) (b). Effects of season on the number of vascular loops in the ovine hypophysial stalk. ***p < 0.01. Adapted from Castle‐Miller et al. (2017).

Temporal changes in the vascular arrangements of the gland likely play a vital role in the physiological adaptation to the environment. Indeed, plasticity in the vascular loops of the hypophysial stalk of rodents has been reported under normal physiological conditions in response to external cues. In mice, food deprivation was shown to induce a structural reorganization of tanycyte tight‐junctions and an increase in the fenestration and permeability in the capillary loops that collectively enhanced the transport of homeostatic signals as an adaptive response to fasting (Langlet et al., 2013). Similarly, in this species the hypophysial microvasculature was shown to adapt to the needs of adjacent endocrine cells as part of the response to a physiological demand (Mollard et al., 2012). In seasonally breeding species, the interaction between the hypophysial cavernous sinus and the carotid rete, which allows the counter‐current transfer of substances from venous blood to arterial blood supplying the brain, was shown to be affected by stage of the annual reproductive cycle in the sheep, with no blood exchange during seasonal anoestrus (summer) (Krzymowski et al., 1992), and in the songbird vascular plasticity in specific brain regions was also reported across seasons (Chen et al., 2013). Notably, dynamic responses to time of year were recorded in the vascular architecture of the ovine hypophysial stalk. These comprised a remarkable increase in the number of vascular loops connecting the pars tuberalis with the infundibulum, and an increase in loop surface area, but not in loop length, during the long days of summer (ovine nonbreeding season) compared to the short days of winter (ovine breeding season) (Figure 1), and this was associated with a significant increase in endothelial cell proliferation at this time of year (Castle‐Miller et al., 2017).

This finding revealed that temporal changes in this vascular system of the hypophysial stalk could readily increase or decrease the delivery of hypothalamic stimulatory and inhibitory signals to the endocrine cells of the gland as part of the mechanisms that control the seasonal output of endocrine systems that show overt circannual oscillation such as lactotrophs, gonadotrophs, and thyrotrophs. This mechanism of adaptation could operate in conjunction with other angiogenic related mechanisms, such as the dynamic retraction and protraction of “endfeet” processes of tanycytes (Prevot et al., 1999), which has been shown to regulate the interaction between the fenestrated capillaries of the median eminence and hypothalamic axonal terminals and play a role in the seasonal reproduction of birds (Yamamura et al., 2004). In support of the findings demonstrating seasonal plasticity in the hypophysial microvasculature, a recent study has provided evidence for a seasonal regulation of the vascular supply to the arcuate nucleus of the ovine hypothalamus, with an increase in the vascular density during the long days of summer (Chevillard et al., 2022).

**Figure 2:**
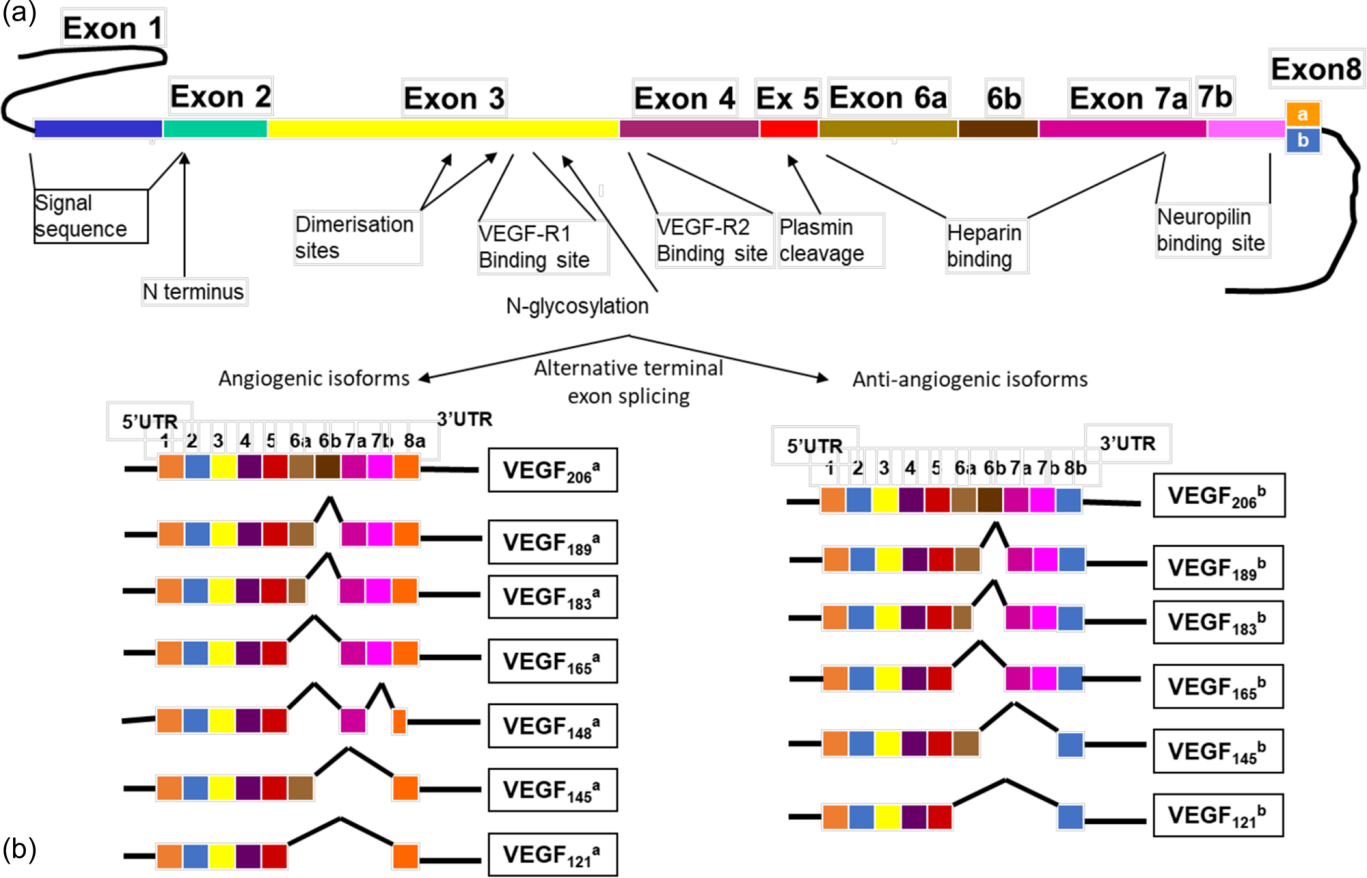
Diagrammatic representation of the structure of the *VEGF *gene and vascular endothelial growth factor (VEGF‐A) splice variants (a). Structure of the *VEGF* gene denoting the exon 8 alternative splice sites (b). Proangiogenic (VEGF‐A_xxx_a) and antiangiogenic (VEGF‐A_XXX_b) families of VEGF‐A isoforms. Adapted with permission from Nowak et al. (2008).

As for other organs, angiogenesis and vascular endothelial cell function in the hypophysis are under VEGF regulation (Bates et al., 2018). First identified by Folkman in 1971 as a controller of tumor growth, VEGFs are a family of peptides that comprise five members (VEGF‐A, VEGF‐B, VEGF‐C, VEGF‐D, and placental growth factor), each encoded by five distinct multiexon genes, of which VEGF‐A plays the major role in angiogenesis and vascular permeability (Bates et al., 2018; Dvorak et al., 1995; Ferrara & Davis‐Smyth, 1997). Alternative splicing of the gene encoding VEGF‐A in exons 6 and 7 leads to proteins of different length (e.g., 120, 164, or 188 aa in sheep, equivalent to 121, 165, and 189 aa in humans) (Bates et al., 2002) of which VEGF‐A_164_/VEGF‐A_165_ has been the most studied. Critically, two families of VEGF‐A isoforms have been identified, one that promotes angiogenesis and vascular permeability referred to as VEGF‐A_xxx_a (where xxx denotes the number of amino acids, and a/b denotes the carboxy terminal amino acid sequence) and an antiangiogenic, antivascular permeability family referred to as VEGF‐A_xxx_b (Bates et al., 2002). The two families result from alternative splicing of the gene in exon 8, where use of the proximal splice site leads to the generation of the proangiogenic VEGF‐A_xxx_a isoforms, and use of the distal splice site leads to the generation of the antiangiogenic VEGF‐A_xxx_b isoforms (Bates et al., 2002; Nowak et al., 2008) (Figure 2). It has been demonstrated that the remodeling of the vascular loops of the pars tuberalis results from differential production of the two isoform families of VEGF‐A in the ovine hypophysis at opposite times of the year (Castle‐Miller et al., 2017). Indeed, the antiangiogenic VEGF‐A_164_b isoform was shown to be upregulated in the breeding season during the short days of winter, leading to a reduction in the number of vascular loops at that time of year, whereas the proangiogenic VEGF‐A_164_a isoform was upregulated in the nonbreeding season during the long days of summer, leading to the significant increase in the number of vascular loops at this stage of the annual physiological cycle. In rodents, other external cues, such as feeding/fasting, have been shown to induce alternative splicing of genes in a tissue specific manner (McGlincy et al., 2012). It is important to note that no differences in ovine hypophysial VEGF‐A were found between breeding season and nonbreeding season animals when the expression was assessed using an antibody that detects total VEGF‐A content, and therefore does not differentiate amongst specific isoforms (Castle‐Miller et al., 2017), which corroborated previous findings in this species (Jabbour et al., 1997); this highlighted that the total output of hypophysial VEGF‐A is not affected by time of year, but that the specific and differential production of pro and antiangiogenic variants is seasonally regulated to induce the required microvascular adaptation to the annual changes in the environment. This appears to be a conserved mechanism of adaptation as differential expression of VEGF‐A isoforms has also been detected in the horse, a long day breeder, across seasons (Yeomans et al., 2014).

VEGFs can act on two type of receptor families, the VEGF receptor (VEGFR) family that comprises VEGFR1, VEGFR2, and VEGFR3, and the neuropilin family that comprises Neuropilin 1 and Neuropilin 2; because neuropilins are able to regulate the activity of ligand induced VEGFR signaling they are also known as coreceptors (Bates et al., 2018). Binding of the proangiogenic VEGF‐A_165_a variant to VEGFR2 on endothelial cells causes robust receptor phosphorylation and triggers a complex intracellular signaling cascade to induce angiogenesis, increase vascular permeability and promote vasodilation (Koch et al., 2011); conversely, binding of the antiangiogenic VEGF‐A_165_b variant to VEGFR2 causes little phosphorylation (Kawamura et al., 2008) and does not activate Neuropilin 1 (Cebe Suarez et al., 2006), failing to trigger the full signaling cascade necessary to induce angiogenesis, increase vascular permeability and promote vasodilation (Oltean et al., 2015; Woolard et al., 2004). Therefore, although VEGF‐A_165_a and VEGF‐A_165_b compete and bind to the same receptor with the same affinity, their pro and antiangiogenic effects counteract each other (Harper & Bates, 2008; Woolard et al., 2004). This implies that mechanisms regulating the differential production pro and antiangiogenic VEGF‐A variants within the pituitary are of critical importance in the physiological adaptation to predictable annual environmental variations.

Interestingly, VEGF‐A was first isolated and purified from FS cells derived from the bovine pituitary (Ferrara & Hanzel, 1989). Because these cells not only are a major source of VEGF‐A in this organ but also contain VEGFR2, an autocrine mode of control of FS cells by their own production of VEGF‐A is warranted. Seasonal plasticity in this mechanism of autocrine regulation in the FS cell network could thus contribute to underlie circannual changes not only in the pituitary microvasculature but also in hormone output through paracrine effects on endocrine cells expressing VEGFR2. Indeed, seasonal changes in the content of VEGFR2 in the FS cell population and lactotroph cells have been observed in the ovine pituitary pars distalis, with a significant increase in the nonbreeding season during the long days of summer, concomitantly with the enhancement in proangiogenic VEGF‐A_164_a expression and endothelial cell proliferation at this time of year (Castle‐Miller et al., 2017). In addition, dynamic morphological changes in the FS cells have been reported in several species throughout different stages of their circannual physiological cycles (Acosta et al., 2010; Christian et al., 2015; Vitale et al., 2001). In sheep, intracellular structural modifications that included an enlarged cytosol area and less condensed nuclear chromatin, together with significant increases in adherent junctions and in the elongated “stellate‐like” processes surrounding endocrine cells were detected in anoestrous animals during the long days of summer (Christian et al., 2015). Therefore, the seasonally‐induced differential expression of hypophysial VEGF‐A isoforms along with the circannual changes in VEGFR2 expression in FS cells point to a critical role of VEGF‐A in the temporal plasticity of the FS system across the annual physiological cycle, contributing to control microvascular remodeling further through the regulation of its own production.

## 3 MELATONIN REGULATES CIRCANNUAL DIFFERENTIAL PRODUCTION OF HYPOPHYSIAL VEGF‐A ISOFORMS

Adaptation to seasonal changes in the environment requires the ability to measure time of year to predict or anticipate the conditions of the incoming season and synchronize circannual physiological cycles in reproduction, metabolism, body weight, fat deposition, pelage molt, and many other biological traits to the impending local conditions to ensure the survival of the species. In mammals living in temperate zones, the annual cycle in day length (photoperiod) is the primary external cue that entrains the aforementioned physiological rhythms to coordinate the phase of the innate rhythm to the correct season (Lincoln, 2006a). Photoperiodic information is transduced through the 24‐h pattern of melatonin secretion from the pineal gland (Goldman, 2001; Ravault & Thimonier, 1988; Tamarkin et al., 1985). Because the secretion of melatonin is increased at night, the duration of the nocturnal melatonin output provides an index of night length (and by subtraction of day length) and consequently of time of year, with long nights in the short days of winter producing a long melatonin signal and short nights in the long days of summer producing a short melatonin signal (Bartness et al., 1993; Bittman et al., 1983; Hastings, 1996). This duration‐based, nocturnal melatonin transducer of photoperiodic information results from two separate mechanisms that work together to produce the decoding system (Schwartz et al., 2009). Photoreceptors in the retina detect the light signal and transform it into a neural signal that through a retino‐hypothalamic‐sympathetic pathway reaches the pineal gland and suppresses melatonin synthesis (Ganguly et al., 2002; Klein et al., 1997; Sugden, 1991). In addition to this potent inhibitory effect of light on melatonin output, the daily pattern of melatonin secretion is controlled by a distinctive region of the suprachiasmatic nucleus (SCN) of the hypothalamus, the central circadian pacemaker, which is entrained by the 24‐h light:dark cycle through a monosynaptic pathway from the retina (Illnerova & Sumova, 1997; Sumova et al., 1995); this is why under constant darkness an entrained SCN continues to induce a circadian rhythm of melatonin output (Earl et al., 1990; Liu & Borjigin, 2005). The relative importance of the two mechanisms of control on melatonin synthesis varies across species (Stehle et al., 2001), but the masking, inhibitory effects of light potently suppress melatonin output in ungulates (Tortonese & Short, 2012; Tortonese et al., 2011).

Melatonin nocturnal secretion acts in the brain and the pituitary gland to translate the effects of photoperiod on annual physiological cycles (Lincoln, 2006b). In sheep, microimplants of melatonin in the medio‐basal hypothalamus (Tortonese & Lincoln, 1995) and in the premammillary hypothalamic region (Malpaux et al., 1998) affected gonadotrophin secretion, whereas in the Syrian hamster lesions in the melatonin sensitive dorsomedial hypothalamic nucleus prevented the specific response to melatonin infusions on the reproductive axis (Maywood et al., 1996). The vast majority of melatonin MT1 receptors, however, are expressed in the hypophysial pars tuberalis (Reppert et al., 1994). Before the cloning of the MT1 receptor, high density of melatonin binding sites had been identified in this region of the rat (Williams & Morgan, 1988) and sheep (de Reviers et al., 1989; Morgan et al., 1989) pituitaries, a finding that was then corroborated in other species (Masson‐Pevet & Gauer, 1994). These receptors were demonstrated to be functional, as they were shown to mediate the photoperiodic regulation of prolactin secretion and, specifically, the suppressive effects of melatonin on prolactin output (Lincoln & Clarke, 1994). Recent studies have revealed that the pars tuberalis‐specific cells responsive to melatonin in the ovine pituitary produce VEGF‐A and that melatonin MT1 receptors are also expressed in the vascular loops connecting the pars tuberalis with the infundibulum (Castle‐Miller et al., 2017). The functional significance of this finding in the photoperiodic signal transduction mechanism that regulates seasonal angiogenesis in this tissue was investigated both “in vitro” and “in vivo.” Using a primary cell culture paradigm where ovine pars tuberalis cells were daily exposed to melatonin treatments mimicking a summer night signal (short duration, 8 h/day) or a winter night signal (long duration, 16 h/day) over 6 days, it was found that the VEGF‐A isoform production was differentially regulated by the melatonin signals. Because both seasonally matching and nonmatching melatonin signals were used in cultures generated in each season, the strategy allowed the differentiation of the effects of melatonin from those resulting from its interaction with the circannual phase. The short duration melatonin signal characteristic of the long days of summer induced upregulation (both of gene expression and protein output) of the proangiogenic VEGF‐A_164_a variant in cultures generated both in the summer and in the winter. Conversely, the long duration melatonin signal characteristic of the short days of winter induced upregulation (both of gene expression and protein output) of the antiangiogenic VEGF‐A_164_b variant, and this also occurred in cultures generated both in the summer and in the winter. In each season, the effect of the matching melatonin regimen on the differential secretion of VEGF‐A isoforms was detected on the first day, whereas for the nonmatching regimen the effect became significantly apparent on the third day, revealing the requirement of the pars tuberalis cells to entrain to the new melatonin signal. This melatonin duration‐dependent differential VEGF‐A isoform production was corroborated “in vivo” in sheep exposed to natural long days and treated with exogenous melatonin. In this study, melatonin timely administered to extend the duration of the natural nocturnal peak to 16 h/day, significantly upregulated the expression of the antiangiogenic VEGF‐A_164_b isoform in the pituitary (I. Clarke, D. Bates, and D. Tortonese, unpublished results). These antiangiogenic effects of melatonin were corroborated in a recent study where splicing‐sensitive fluorescent reporters were used for the screening of VEGF‐A modulators (Star et al., 2021). How the duration of melatonin exposure induces differential output of VEGF‐A variants is still unclear, but is likely to involve the regulation of the slicing‐factor kinase SRPK1 and selective activation of the splicing factors SRSF1 or SRSF6 which are known to control the alternative splicing of the *VEGF* gene in exon 8 at the proximal and distal splice sites, respectively, for the corresponding production of pro or antiangiogenic VEGF‐A variants (Amin et al., 2011; Bowler & Oltean, 2019; Nowak et al., 2008). As the pars distalis is deprived of melatonin receptors (Williams, 1989), and the differential expression of VEGF‐A isoforms was shown to be melatonin duration‐dependent, the upregulation of the antiangiogenic VEGF‐A_164_b isoform that was also detected in the pars distalis is likely to result from the previously described VEGF‐A control of the FS cell network. In rodents, melatonin induces senitization of the adenosine A2b receptor with subsequent increase in cAMP and cAMP response element binding protein phosphorylation (von Gall et al., 2002), and in aortic smooth muscle cells adenosine and cAMP have been shown to stimulate VEGF‐A expression (Pueyo et al., 1998), indicating that the effects of melatonin on VEGF‐A isoform production are predictably downstream of that signaling pathway. Collectively, the results of these studies show that a melatonin duration‐based mechanism within the pituitary gland underlies the differential production of VEGF‐A isoforms to regulate angiogenesis locally within this organ. The resulting temporal remodeling of the vascular connection between the photoperiod‐sensitive pars tuberalis and the FS cell‐rich infundibulum, will increase or decrease the humoural communication between the brain and the pituitary throughout the annual physiological cycle as part of an adaptive strategy to the changing environment.

## 4 MELATONIN INDUCED DIFFERENTIAL EXPRESSION OF VEGF‐A ISOFORMS IN THE PARS TUBERALIS DECODES PHOTOPERIODIC REGULATION OF SEASONAL ENDOCRINE OUTPUT

**Figure 3:**
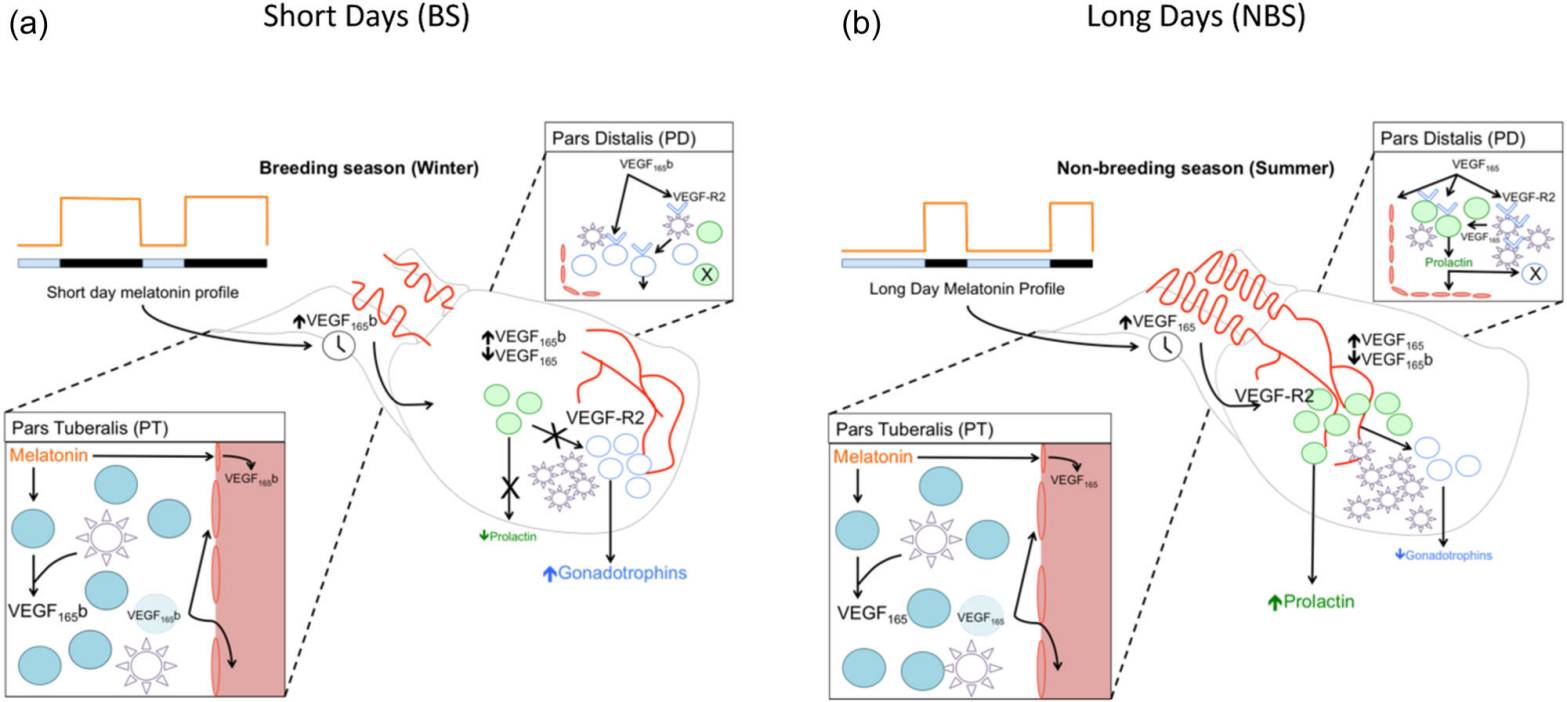
Photoperiodic signal transduction relay underlying hypophysial control of circannual prolactin and gonadotrophin (follicle stimulating hormone ‐FSH) cycles in sheep. Pineal melatonin secretion translates the effects of photoperiod on the seasonal secretion of prolactin and FSH by evoking differential synthesis and release of proangiogeneic vascular endothelial growth factor (VEGF‐A_164/165_a) and antiangiogenic (VEGF‐A_164/165_b) isoforms in the pars tuberalis and vascular loops of the hypophysial stalk to regulate seasonal physiology (a). In the winter (ovine breeding season ‐BS), the long duration of melatonin exposure that mirrors the long nights at this time of year upregulates VEGF‐A_164/165_b, leading to a reduction in angiogenesis and in the density of VEGF receptors in endocrine and folliculo‐stellate (FS) cells of the pars distalis, with suppression of prolactin output, and no inhibition of FSH (b). In summer (ovine non‐BS, NBS), the short duration of melatonin exposure that mirrors the short nights at this time of year upregulates the secretion of VEGF‐A_164/165_a, resulting in an increase in angiogenesis and in the density of VEGF receptors in endocrine and FS cells of the pars distalis, with stimulation of prolactin secretion, and inhibition of FSH output. Thus, an angiogenesis‐based intra‐hypophysial system contributes to decode the circannual photoperiod/melatonin signal to regulate the endocrine adaptation to the predictable changes in the environment. From Castle‐Miller et al. (2017).

In a pioneering study using hypothalamo‐pituitary‐disconnected (HPD) sheep, an animal model in which the hypothalamus is surgically disconnected from the pituitary through removal of the arcuate nucleus and median eminence without compromising the vascular supply to the gland (Clarke et al., 1983), long‐term changes in prolactin secretion continued to be driven by photoperiod, with high concentrations under long days (16 h light:8 h dark) and low concentrations under short days (8 h light:16 h dark) similar to sham operated controls, and exogenous melatonin potently suppressed high prolactin concentrations during long days in both groups (Lincoln & Clarke, 1994). In this animal model, the photoperiodic control of other endocrine systems is blocked due to removal of hypothalamic axonal terminals of stimulatory and inhibitory systems in the median eminence, but the melatonin‐mediated photoperiodic regulation of circannual cycles in prolactin is maintained, and this occurs in the absence of inhibitory dopaminergic input that controls prolactin homeostasis in all mammals (Lincoln & Clarke, 2002). These findings provided convincing proof for a key role of the pars tuberalis melatonin MT1 receptors in the circannual regulation of an endocrine system that is involved in the adaptive responses of a variety of traits such as molting, metabolism, stress, and sexual behavior. Critically, this photoperiodic regulation of prolactin requires an intra‐pituitary paracrine mode of communication because the melatonin receptor‐rich pars tuberalis of the ovine pituitary is devoid of lactotrophs (Gross, 1984), and the lactotroph‐rich pars distalis is devoid of melatonin receptors (Williams, 1989). This conundrum has led to an inexorable search for the pars tuberalis effector of the photoperiodic signal transduction relay. Several compounds secreted by the pars tuberalis such as the tachykinins neurokinin A and substance P are known to stimulate prolactin secretion (Dupré et al., 2010; Eckstein et al., 1980; Skinner, 2009), and the production of the endocannabinoid 2‐arachidonoylglycerol, which can stimulate prolactin indirectly through a mediatory cell type, was shown to be influenced by photoperiod (Korf, 2018). Moreover, unidentified different‐sized proteins distinctive to the pars tuberalis specific cells were identified in the bovine pituitary (Guerra & Rodriguez, 2001). However, whether the output of any of those pars tuberalis products is affected by the duration of the nocturnal melatonin signal to mediate the photoperiodic control of prolactin secretion has not yet been resolved. Pioneering work by Wittkowski et al. (1988) showed that the immunohistochemical expression of a TSH‐like protein in the pars tuberalis of the Siberian hamster was modified by changes in photoperiod, and subsequent work by this group demonstrated that these photoperiodic effects on the expression of the common alpha and TSH beta subunits were prevented by pinealectomy (Bockmann et al., 1996). Similarly, the expression of the *TSH* beta subunit messenger RNA (mRNA) in the pars tuberalis specific cells of the sheep was reported to be driven by photoperiod (Hanon et al., 2008). However, the original study by Gross (1984) had shown that the pars tuberalis of this species does not contain thyrotrophs, a finding that when combined with the observed disproportion between gene and protein expressions for the alpha and beta subunits led Wittkowski et al. (1999) to conclude that “TSH cannot be regarded as the major product of pars tuberalis specific cells.” The conclusion was further supported by the absence of TSH immunoreactivity in the sheep pars tuberalis under either short days (breeding season) or long days (nonbreeding season) using a specific ovine TSH antibody that detected abundant expression of thyrotrophs in the pars distalis (Hodson et al., 2013). Similarly, the pars tuberalis specific cells of the bovine pituitary were shown to express the common alpha subunit but not any of the hormones secreted by the pars distalis (Guerra & Rodriguez, 2001). The apparent discrepancy/controversy was untangled by further molecular studies showing that, although the structure of the alpha and TSH beta subunits of the pars tuberalis specific cells appear to be the same as those of the TSH of pars distalis origin, the pars tuberalis cells producing the TSH product do not contain thyrotrophin‐releasing hormone (TRH) receptors or the transcription factor Pit1 and do not respond to T3 regulation, revealing that these cells are structurally and functionally different from pars distalis thyrotrophs (Bockmann et al., 1997). A comprehensive study in mice by Ikegami et al. (2014) reported that tissue specific glycosylation in pars tuberalis and pars distalis thyrotrophs leads to molecules of starkly different biological activities, and that in this nonphotoperiodic species exogenous melatonin suppressed TSH of pars tuberalis origin. The involvement of the thyroid axis in the regulation of circannual biological rhythms had been demonstrated in sheep by experiments showing that, when performed in the autumn, thyroidectomy blocked the termination of the breeding season in the following spring and allowed the ewes to continue to exhibit estrous cycles in the nonbreeding season; importantly, this effect was counteracted by thyroxine replacement (Karsch et al., 1995; Moenter et al., 1991; Nicholls et al., 1988). However, whether the aforementioned reported changes in pars tuberalis *TSH* mRNA in response to photoperiodic manipulations are mirrored by changes in the translated secreted protein to mediate the effects of photoperiod on the circannual rhythm of prolactin remains to be determined, as no measurements of pars tuberalis TSH output have been reported in this species. Indeed, to our knowledge, the secretion of TSH from the pars tuberalis of the sheep has not been determined either in the pituitary portal circulation, through the well‐established method of blood sampling from these vessels developed by Clarke and Cummins (1982), or in pars tuberalis primary cultures or explants exposed to different duration of melatonin treatments. It is possible that the described changes in the pars tuberalis *TSH* mRNA expression and those in tachykinins and endocannabinoids production are part of a collective response to photoperiodism, but whether these changes are involved in the photoperiodic control of an endocrine output from the pars distalis, such as the seasonal prolactin cycle, remains to be ascertained. Notwithstanding the potential involvement of these products, or of any still unidentified substance, in this paracrine mechanism, it has been shown that the melatonin‐duration induced differentially secreted VEGF‐A isoforms from the ovine pars tuberalis in the long days of summer and short days of winter act as paracrine factors in the pars distalis to regulate the annual pattern of prolactin (Castle‐Miller et al., 2017). Moreover, the differentially secreted VEGF‐A variants in response to the melatonin duration signal also affect seasonal gonadotrophin secretion. Specifically, the upregulation of the proangiogenic VEGF‐A variants prompted by the short duration of the melatonin signal during the long days of summer stimulates prolactin secretion, inhibits FSH secretion and increases the density of VEGF‐A receptors in endocrine and FS cells of the pars distalis, whereas the upregulation of the antiangiogenic VEGF‐A variants prompted by the long duration of the melatonin signal in the short days of winter does not stimulate prolactin secretion, stimulates FSH secretion and reduces the density of VEGF‐A receptors in endocrine and FS cells of the pars distalis (Castle‐Miller et al., 2017). Thus, the melatonin duration‐induced differential production of VEGF‐A isoforms in the pars tuberalis constitutes part of the photoperiodic signal transduction relay that underlies the circannual prolactin and gonadotrophin cycles (Figure 3). As VEGF‐A receptors are not only expressed in lactotrophs but also in FS cells, and FS cells possess intimate contacts not only with lactotrophs and gonadotrophs but also with the remaining three endocrine cell types of the pars distalis, that is, corticotrophs, somatotrophs, and thyrotrophs, the VEGF‐A isoform response to the photoperiod/melatonin signal can indirectly affect these other endocrine axes to regulate, for example, the seasonal response to stress and the temporal changes in metabolism as part of the adaption to annual changes in the environment. In addition, because the gonadotrophs selectively express prolactin receptors (Tortonese et al., 1998), the differential VEGF‐A isoform response to melatonin will contribute to regulate seasonal fertility also indirectly through its control of the lactotrophic axis.

## 5 HYPOPHYSIAL VEGF‐A SYSTEMS AND CIRCANNUAL TIME MEASUREMENT

The melatonin duration signal that translates photoperiodic timing in the pars tuberalis specific cells is known to regulate the circadian expression of clock genes in this tissue. The transcriptional activators (CLOCK and BMAL) and transcriptional inhibitors (Per1, Per2 and Cry1, Cry2) of the molecular clockwork show distinctive 24‐h rhythms of gene expression with amplitudes and timings different from those recorded for the same genes in the SCN (Hazlerigg et al., 2004; Lincoln et al., 2002). Critically, the regulatory role of the melatonin signal on the timing of clock gene expression is apparent in the pars tuberalis but not in the SCN (Hazlerigg et al., 2004; Messager et al., 2001; von Gall et al., 2002, 2005). Within the pars tuberalis, the expression of *Cry1* peaks at the beginning of the dark phase (Lincoln et al., 2002), whereas the expression of *Per1* peaks at the beginning of the light phase (Lincoln et al., 2002; Messager et al., 2001). In the Syrian hamster, melatonin suppressed the peak of *Per1* and pinealectomy abolished it (Messager et al., 2001). In sheep, impairment of the rise of melatonin at the beginning of the dark phase delayed the increase in *Cry1* expression, whereas exogenous melatonin given to match the rise of its nocturnal peak stimulated *Cry1* expression (Hazlerigg et al., 2004). Similarly, in the MT1 melatonin receptor knock out mouse model the nocturnal peak of *Cry1* mRNA is not expressed (von Gall et al., 2005). The gap between the peak of *Cry1* induced by the increase in melatonin at the beginning of the night and the peak of *Per1* induced by the decline of melatonin at the beginning of the day provides an indication of the duration of the night and will determine the level of CRY/PER protein heterodimer formation in the cytoplasm that will then translocate to the nucleus to operate as transcription factors to inhibit BMAL1 and CLOCK transcription (Lincoln, 2006b). The *Cry1*/*Per1* interval of the circadian molecular clockwork in the pars tuberalis has thus been proposed to play a key role in the decoding of the photoperiod/melatonin signal (Lincoln, 2006b), but whether this mechanism is involved in the melatonin‐induced differential expression of VEGF‐A isoforms within the pituitary has not yet been resolved.

Under constant photoperiodic conditions a state of photo‐refractoriness develops in both short‐ and long‐day seasonal breeders and endogenous biological rhythms are freely expressed, which points to the existence of a circannual clock undertaking the control of these rhythms. In sheep exposed to constant long days, the secretion of prolactin was shown to display circannual cycles even though the melatonin signal and the pars tuberalis clockwork faithfully continued to reflect the long day photoperiod (Lincoln et al., 2005). Similarly, in Syrian hamsters, prolonged exposure to short days resulted in an increase in prolactin despite the melatonin signal and the clock gene expression in the pars tuberalis continued to track the short‐day photoperiod (Johnston et al., 2003). The pars tuberalis has been identified as the site of the circannual biological clock (Korf, 2018; Lincoln et al., 2006; Wood & Loudon, 2018). Part of this conclusion is derived from studies using the ovine HPD model, where animals exposed to constant long days showed a consistent free running prolactin cycle (Lincoln & Clarke, 2000) with a periodicity of approximately 10 months (Lincoln, 2006a). The possible participation of angiogenic mechanisms in the circannual clockwork was investigated in photorefractory Siberian hamsters exposed to constant short days; in these animals, the VEGF‐A output was reverted to that shown under long days (T. Stevenson, N. Reyes Prieto, D. Bates, and D. Tortonese, unpublished results). These findings point not only to the likely involvement of the VEGF‐A system in circannual time measurement, but also to their relative independence from circadian clock gene regulation.

## 6 CONCLUSION

The implication of hypophysial angiogenic systems in photoperiodic timing is crucial for adaptation to seasonal changes in the environment as do they not only regulate the temporal vascular connection between the brain and the hypophysis, but also operate as paracrine communicators transducing the melatonin signal from the pars tuberalis to the pars distalis to ensure the appropriate endocrine output for each season.

## CONFLICT OF INTEREST

The author declares no conflict of interest.

## Data Availability

Data reported in this manuscript are derived from public domain resources. The data that support the findings presented in this article are available from the corresponding author, (DT), upon reasonable request.
